# Nanoparticles modulate surfactant protein A and D mediated protection against influenza A infection *in vitro*

**DOI:** 10.1098/rstb.2014.0049

**Published:** 2015-02-05

**Authors:** Zofi McKenzie, Michaela Kendall, Rose-Marie Mackay, Teresa D. Tetley, Cliff Morgan, Mark Griffiths, Howard W. Clark, Jens Madsen

**Affiliations:** 1 Child Health, Clinical and Experimental Sciences, Faculty of Medicine, University of Southampton, Southampton General Hospital, Southampton SO16 6YD, UK; 2 School of Metallurgy and Materials, University of Birmingham, Birmingham B15 2TT, UK; 3 National Heart and Lung Institute, Imperial College London, London SW3 6LY, UK; 4 Leukocyte Biology, Imperial College London, Royal Brompton Campus, London SW3 6NP, UK; 5 Institute for Life Sciences, University of Southampton, Southampton SO17 1BJ, UK; 6 National Institute for Health Research, Southampton Respiratory Biomedical Research Unit, Southampton Centre for Biomedical Research, University Hospital Southampton NHS Foundation Trust, Southampton SO16 6YD, UK

**Keywords:** nanoparticles, surfactant, mucosal, innate immunity, collectin, surface chemistry

## Abstract

Numerous epidemiological and toxicological studies have indicated that respiratory infections are exacerbated following enhanced exposure to airborne particulates. Surfactant protein A (SP-A) and SP-D form an important part of the innate immune response in the lung and can interact with nanoparticles to modulate the cellular uptake of these particles. We hypothesize that this interaction will also affect the ability of these proteins to combat infections. TT1, A549 and differentiated THP-1 cells, representing the predominant cell types found in the alveolus namely alveolar type I (ATI) epithelial cells, ATII cells and macrophages, were used to examine the effect of two model nanoparticles, 100 nm amine modified (A-PS) and unmodified polystyrene (U-PS), on the ability of SP-A and SP-D to neutralize influenza A infections *in vitro*. Pre-incubation of low concentrations of U-PS with SP-A resulted in a reduction of SP-A anti-influenza activity in A549 cells, whereas at higher concentrations there was an increase in SP-A antiviral activity. This differential pattern of U-PS concentration on surfactant protein mediated protection against IAV was also shown with SP-D in TT1 cells. On the other hand, low concentrations of A-PS particles resulted in a reduction of SP-A activity in TT1 cells and a reduction in SP-D activity in A549 cells. These results indicate that nanoparticles can modulate the ability of SP-A and SP-D to combat viral challenges. Furthermore, the nanoparticle concentration, surface chemistry and cell type under investigation are important factors in determining the extent of these modulations.

## 1. Introduction

The rapid growth in the nanotechnology industry has led to concerns regarding the potential health implications of nanomaterial exposures. The toxicological impact of nanomaterials is particularly concerning in light of the evidence which shows that airborne particulate matter (PM) is responsible for enhanced morbidity and mortality from cardiopulmonary causes [1–4]. One of the adverse respiratory outcomes following exposure to high PM levels is increased incidence and severity of respiratory infections [5]. This is of particular concern in young children and the elderly [6,7]. Pre-natal exposure to PM_2.5_ has been shown to increase the incidence of recurrent pneumonia and acute bronchitis in children in a concentration dependent manner. Moreover, incidences of these pulmonary infections following exposure to PM_2.5_ were much higher in children with asthma [8]. In children less than 1 year, every hour of exposure to indoor PM_2.5_ above 100 µg m^−3^ led to a 7% increase in the risk of acute lower respiratory infection (ALRI); however, this risk was not found in children of 1–2 years [9]. These indoor exposure concentrations are common in developing countries where ALRI accounts for around one in five of the deaths of children under 5 years [10]. Pre-exposure to more than 33.3 µg m^−3^ PM_10_ has also been shown to delay the resolution of respiratory tract infections by around 20% in healthy infants [5]. Diesel exhaust (DE) and diesel exhaust particulates (DEP) have been shown to enhance and exacerbate influenza A virus (IAV) infections in mice [11–14]. Furthermore, pre-exposure to DE and DEP has been shown to increase respiratory syncytial virus (RSV) infection and viral induced lung inflammation in mice *in vivo*. Interestingly, this study showed a reduction in surfactant protein A (SP-A) expression during RSV infection following exposure to DE [15]. In another study, the exposure of mice to DEP for six months prior to IAV infection resulted in an increased incidence of infection but did not alter mortality in IAV exposed mice [16]. The evidence surrounding the enhanced susceptibility to respiratory infections following particulate exposure relates to ambient particulate matter rather than engineered nanoparticles. However, these studies serve as useful guides to understand the potential toxicological issues which may be faced following exposure to engineered nanoparticles.

SP-A and SP-D are important innate immune molecules found primarily in pulmonary surfactant, the lipoprotein substance found at the air–liquid interface in the alveoli of the lungs. SP-A and SP-D are calcium-dependent lectin pattern recognition molecules and belong to the subfamily known as the ‘collectins’. The pulmonary collectins are oligomerized proteins and play an integral role in the innate immune defence of the lung. They recognize and bind specific carbohydrate moieties on the surface of microorganisms and can act to neutralize microbial challenge via agglutination and opsonization [17–20]. SP-A and SP-D form a part of the defence against IAV infection. SP-A and SP-D bind to haemagglutinin (HA), a surface glycoprotein of IAV; this interaction directly inhibits cellular infection by preventing the interaction of HA with sialic acid containing receptors. SP-A and SP-D bind HA through different mechanisms. HA binds to the partially sialylated asparagine 187 residue in the carbohydrate recognition domain of human SP-A; this interaction is calcium independent and is not antagonized by mannan [21–23]. SP-D binds to glycosylation sites on HA and is classified as a β-type inhibitor of influenza as this interaction is calcium-dependent and SP-D is heat labile and resistant to degradation by neuraminidase (NA) [20]. SP-D is also able to bind to NA, the second surface glycoprotein of IAV, and inhibit the release of progeny virions from infected cells [23,24]. The binding of SP-A and SP-D to IAV also aids the neutralization of IAV through the agglomeration and opsonization of virions [25–27].

SP-A and SP-D have been reported to associate with nanoparticles and this interaction has been shown to alter the cellular uptake of these nanoparticles [28–30]. Metal oxide nanoparticles incubated in bronchoalveolar lavage (BAL) have been shown to adsorb SP-A; the extent of this interaction was shown to be dependent on the particle surface chemistry [31]. The SP-A and SP-D interaction with magnetite particles possessing different polymer coatings has also been investigated. These studies have shown that both SP-A and SP-D enhance the uptake by macrophages of magnetite particles with hydrophobic and hydrophilic surface coatings. However, SP-D enhanced the uptake of hydrophilic nanoparticles to a greater extent than SP-A; whereas for hydrophobic particles the reverse was true [28,29]. Furthermore, it has been shown that SP-D is removed from suspension when co-incubated with carbon black particles [32].

**Figure 1.**
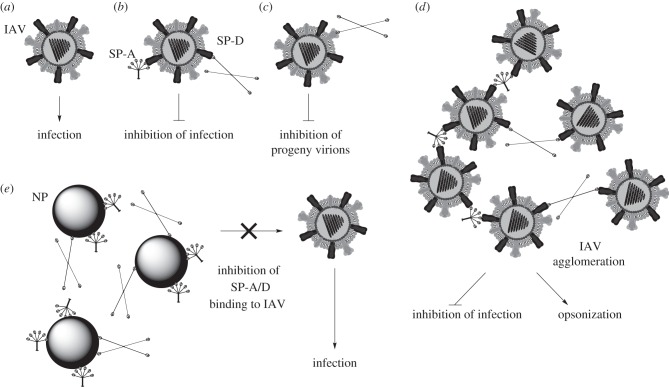
Hypothesis of nanoparticle (NP) sequestration of SP-A and SP-D enhancing IAV infection. (*a*) Influenza A virus (IAV) initiates infection through the binding of the surface glycoprotein haemagglutinin (HA) to sialic acid containing receptors on the host cell. (*b*) Surfactant protein A (SP-A) and SP-D bind to HA and prevent its interaction with sialic acid [20–23]. (*c*) SP-D binds neuraminidase on IAV surface and prevents the release of progeny virions from the host cell following replication [23,24]. (*d*) SP-A and SP-D act to agglomerate IAV which directly inhibits infection and also enhances the opsonization of IAV by professional phagocytes such as macrophages [25–27]. (*e*) It is hypothesized that the interaction of SP-A and SP-D with NPs in the alveolar space will reduce their interaction with and neutralization of influenza virus, and thereby enhance susceptibility to infection.

It was therefore hypothesized that the interaction of SP-A and SP-D with nanoparticles could lead to a deficiency in these innate immune molecules and enhance susceptibility to infection (figure 1). In order to test this hypothesis, an *in vitro* infection model was used to determine the effect of 100 nm unmodified polystyrene (U-PS) and amine modified polystyrene (A-PS) particles on the SP-A and SP-D mediated neutralization of influenza virus. This was conducted in three cell lines, TT1, A549 and differentiated THP-1 cells to reflect the major cell types found in the alveolar epithelium, namely the alveolar type I (ATI) and type II cells and the alveolar macrophages.

## 2. Material and methods

### (a) Protein purification

SP-A and SP-D were purified from human bronchoalveolar lavage (BAL). SP-A was purified using butanol extraction as described previously [33]. SP-D was purified using affinity chromatography and size exclusion chromatography [34].

### (b) IAV propagation and purification

IAV H3N2 X79 was propagated in cell culture using MDCK cells and purified on a discontinuous 30–60% sucrose gradient as previously described [35]. Viral titre was determined using the fluorescent focus assay and measured as fluorescent focus units (FFU) ml^−1^.

### (c) Cell culture

TT1 cells were provided by Prof. T. Tetley, Imperial College London, and were grown in DCCM1 media (Cellseco, Porton Down, UK) containing 10% heat inactivated new born calf serum, 100 units ml^−1^ penicillin and 100 µg ml^−1^ streptomycin, 2 mM l-glutamine (Invitrogen, UK) and 0.5 mg ml^−1^ G418 (Sigma, UK) as described previously [36]. A549 cells were grown in RPMI1640 (Invitrogen, UK) containing 10% fetal bovine serum (FBS; Sigma, UK) and 100 units ml^−1^ penicillin and 100 µg ml^−1^ streptomycin. THP-1 cells were kindly provided by Liku Tezera and were grown in RPMI1640 medium containing 10% heat inactivated FBS (PAA, UK) and 100 units ml^−1^ penicillin and 100 µg ml^−1^ streptomycin. A549 and TT1 cells were routinely subcultured every 2–3 days using trypsin. THP-1 cells grow in suspension and were subcultured by diluting cell suspensions in growth medium every 2–3 days to maintain cell density at around 5 × 10^5^–1.5 × 10^6^ cells ml^−1^.

### (d) Nanoparticles

This study used fluorescent orange 100 nm A-PS (Sigma, UK) and fluorescent green 100 nm U-PS (Polysciences, UK) nanoparticles. The size distribution and zeta potentials of these nanoparticles in Tris-buffered saline (TBS) with calcium has previously been characterized and reported [30,37].

### (e) IAV infection

A549 and TT1 cells (0.3 ml) were seeded at a density of 4.16 × 10^5^ ml^−1^ in 48-well plates (Corning, UK). The cells were incubated for 6 h in relevant growth medium then serum starved for 24 h in serum-free (SF) RPMI. THP-1 cells were suspended at a concentration of 4.16 × 10^5^ cells ml^−1^ in growth medium containing 10 nM phorbol 12-myristate 13-acetate (PMA). Cells were plated in 48-well plates (0.3 ml) and incubated for 72 h at 37°C 5% CO_2_ in a humidified atmosphere. PMA was dissolved in dimethyl sulfoxide which was used as a vehicle control and kept at less than 0.1% in all experiments. All cells were serum starved for 24 h in SF RPMI prior to exposure to nanoparticle/IAV suspensions.

Particles were prepared at three times the final concentration in TBS containing 5 mM calcium. SP-A, SP-D and bovine serum albumin (BSA) were also prepared in TBS with 5 mM calcium at three times the final concentration. Equal volumes of nanoparticle suspension and protein suspensions were added to a 48-well plate, the plate was gently agitated and incubated at 37°C for 1 h. IAV was prepared at three times the final concentration (2.23 × 10^5^ FFU ml^−1^) in TBS with calcium and then added to the nanoparticle and protein suspension. The inoculum was then incubated for a further hour at 37°C.

The medium was removed from the serum starved cells and replaced with the inoculum. The cells were then incubated for 1 h at 37°C 5% CO_2_ in a humidified atmosphere. The cells were washed three times in SF RPMI and the cells were then incubated in fresh SF RPMI (0.5 ml) for 18 h at 37°C 5% CO_2_ in a humidified atmosphere.

Following incubation, the cells were washed with calcium/magnesium-free PBS and trypsinized for 5 min. The trypsin was deactivated by adding a 10-fold excess of complete medium. The cell suspension was centrifuged for 5 min at 400 *g* and the cell pellet was resuspended in 1% formaldehyde in PBS and incubated for 1 h at room temperature. The cells were resuspended in 1 ml PBS then centrifuged at 400 *g* for 5 min. The pellet was then resuspended in 1 ml PBS with 0.3% triton (TrPBS) to permeabilize the cell membranes, the cells were then centrifuged at 400 *g* for 5 min. The cells were resuspended in TrPBS containing 1.5 µg ml^−1^ of mouse anti-influenza A nucleoprotein (Abcam, UK) and incubated for 1 h at room temperature. The cells were washed (2 × 1 ml) in TrPBS by centrifuging the sample at 400 *g* for 5 min. The cells were resuspended in TrPBS containing 1 µg ml^−1^ of goat anti-mouse IgG secondary antibody coupled to allophycocyanin fluorophore and incubated for 1 h at room temperature. The cells were then washed twice in TrPBS as above then resuspended in 100 µl of PBS. The percentage of cells infected with IAV was determined using a FACSAria: 10 000 cells were analysed per sample and each experiment was conducted in duplicate.

### (f) Statistics

Relative infection rates were reported as mean values. Differences between two treatments were determined using the Mann–Whitney *U-*test. *p-*Values less than or equal to 0.05 were considered statistically significant and were determined using the Mann–Whitney *U*-test unless otherwise stated. Infection rates were normalized for each experiment prior to analysis. The ability of the particles to modify surfactant protein neutralization was calculated using the following formula:2.1
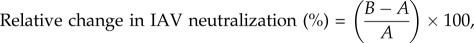
where *B* is the percentage of uninfected cells in the nanoparticle, protein and IAV treatment, and *A* is the percentage of uninfected cells in the appropriate IAV and protein control.

## 3. Results

We have previously shown that SP-A and SP-D interact with nanoparticles and that these interactions can alter particle agglomeration and uptake by phagocytes such as macrophages. Here, we present for the first time the effect of nanoparticles on the function of SP-A and SP-D. The effect of 100 nm cationic A-PS and anionic U-PS nanoparticles on the ability of SP-A/SP-D to neutralize IAV infection *in vitro* was examined in TT1, A549 and differentiated THP-1 cell lines. These cells represent the predominant cell types found in the alveolus, namely ATI epithelial cells, ATII cells and macrophages [38].

In this study, concentrations of 5 µg ml^−1^ SP-A and 0.4 µg ml^−1^ SP-D were chosen which were shown to induce a submaximal inhibition of IAV infection (data not shown). Thus any nanoparticle induced modulation in the ability of these proteins to neutralize infection could be detected. Nanoparticles were initially pre-incubated with proteins for 1 h, then influenza was added to the inoculum and incubated for a further hour. This procedure was chosen in order to test the hypothesis that nanoparticles would sequester surfactant proteins and thereby attenuate their ability to neutralize infection. Incubations were conducted in TBS containing 5 mM calcium, sufficient for the lectin functionality of the collectins. This buffer was shown not to alter cell viability or influenza infection rates compared with SF media (data not shown). The nanoparticles used in these experiments have previously been thoroughly characterized [30,37].

### (a) Effect of U-PS on IAV infection

**Figure 2.**
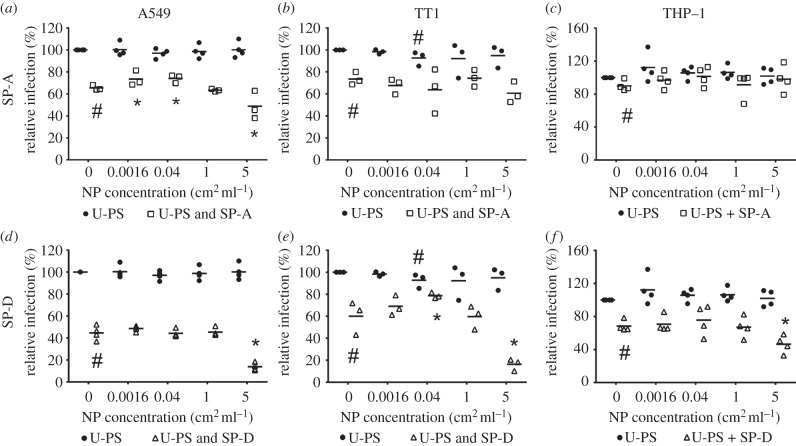
Effect of 100 nm U-PS on SP-A and SP-D mediated neutralization of influenza A infection. U-PS were pre-incubated with 5 µg ml^−1^ SP-A prior to incubation with IAV and treatment of (*a*) A549, (*b*) TT1 and (*c*) differentiated THP-1 cells. U-PS particles were pre-incubated with 0.4 µg ml^−1^ SP-D prior to incubation with IAV and treatment of (*d*) A549, (*e*) TT1 and (*f*) THP-1 cells. Horizontal line represents mean of at least three independent experiments conducted in duplicate. Statistics determined using Mann–Whitney *U*-test where *p ≤ 0.050 compared to relative infection in particle free protein control; #*p* ≤ 0.050 versus IAV alone.

The effects of U-PS at concentrations of 0.0016–5 cm^2^ ml^−1^ (0.0028–8.8 µg ml^−1^) on IAV infection in the three cell types are summarized in figure 2 and the electronic supplementary material, table S1. The pre-incubation of 100 nm U-PS with IAV had no significant effect on IAV infection in A549 and differentiated THP-1 cells at any of the nanoparticle concentrations studied. In TT1 cells, the pre-incubation of 100 nm U-PS particles (0.04 cm^2^ ml^−1^) with IAV resulted in a significant 7.3% reduction in infection compared with IAV alone (*p* = 0.037).

### (b) Effect of U-PS on SP-A/D-mediated IAV neutralization

In A549 cells, U-PS particles had differential effects at high and low doses on the ability of SP-A to neutralize IAV infection (figure 2*a*–*c*). There were significant 8.0 and 8.6% increases in relative infection rates in A549 cells following pre-incubation of SP-A with 0.0016 and 0.04 cm^2^ ml^−1^ U-PS compared with SP-A in the absence of particles (*p* = 0.050). These differences represented 23.2 and 24.9% relative decreases in the efficacy of SP-A to neutralize IAV infection (see equation (2.1)). The pre-incubation of 5 cm^2^ ml^−1^ U-PS with SP-A resulted in a significant 16.7% reduction in IAV infection compared with the IAV and SP-A control in A549 cells (*p* = 0.050). This represented a 48.4% increase in the antiviral activity of SP-A.

In TT1 cells, U-PS particles also had differential effects at high versus low concentrations on SP-D-mediated IAV neutralization (figure 2*e*). The pre-incubation of 0.04 cm^2^ ml^−1^ U-PS with SP-D resulted in a significant 18.7% increase in IAV infection compared with the SP-D and IAV control (*p* = 0.050). This represented a 46.9% decrease in the ability of SP-D to neutralize IAV infection in this cell line. The highest concentration of U-PS studied (5 cm^2^ ml^−1^) resulted in a significant 43.8% reduction in relative infection rates compared with the absence of U-PS (*p* = 0.050). This signifies a 109.8% relative increase in the inhibition of infection rates compared with SP-D.

In A549 and THP-1 cells, the pre-incubation of 5 cm^2^ ml^−1^ U-PS with SP-D resulted in significant 30.8 and 22% reductions in infection compared with SP-D and IAV alone (*p* = 0.021). These represent 55.7 and 69.6% relative increases in the inhibition of IAV infection over SP-D alone (figure 2*d*,*f*).

### (c) Effect of A-PS on IAV infection

**Figure 3.**
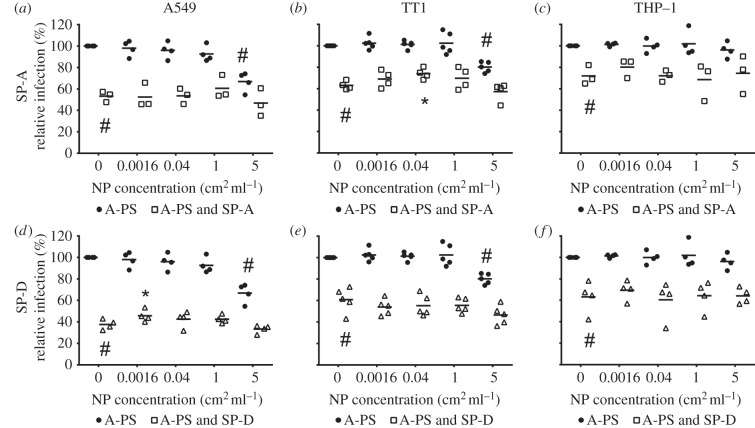
Effect of 100 nm A-PS on SP-A and SP-D mediated neutralization of influenza A infection. A-PS were pre-incubated with 5 µg ml^−1^ SP-A prior to incubation with IAV and treatment of (*a*) A549, (*b*) TT1 and (*c*) differentiated THP-1 cells. A-PS particles were pre-incubated with 0.4 µg ml^−1^ SP-D prior to incubation with IAV and treatment of (*d*) A549, (*e*) TT1 and (*f*) THP-1 cells. Horizontal line represents mean of at least three independent experiments conducted in duplicate. Statistics determined using Mann–Whitney *U*-test where *p ≤ 0.050 compared to relative infection in particle free protein control; #*p* ≤ 0.050 versus IAV alone.

The effects of A-PS on IAV infection are summarized in figure 3 and the electronic supplementary material, table S2. In A549 and TT1 cells, 100 nm A-PS at 5 cm^2^ ml^−1^ resulted in significant 33.2 and 19.8% reductions in IAV infection (*p* = 0.014 and *p* = 0.005).

### (d) Effect of A-PS on SP-A/D-mediated IAV neutralization

In TT1 cells, 0.04 cm^2^ ml^−1^ A-PS particles pre-incubated with SP-A resulted in an 11% significant increase in IAV infection compared with SP-A in the absence of particles (figure 3*b*). This represents a 29.7% reduction in the antiviral efficacy of SP-A, whereas in A549 cells, pre-incubation of 0.0016 cm^2^ ml^−1^ A-PS with SP-D resulted in a 8.1% increase in IAV infection compared with the SP-D control (figure 3*d*). This difference was statistically significant with an associated *p*-value of 0.043 and represents a 13.0% reduction in the capability of SP-D to neutralize IAV infection.

### (e) Effect of bovine serum albumin and nanoparticles on IAV infection

BSA was used to determine the effect of a non-specific protein corona on influenza infection in these experiments. In A549, TT1 and THP-1 cells, the pre-incubation of BSA with 0.0016–5 cm^2^ ml^−1^ U-PS or A-PS had no significant effect on IAV infection compared with the protein and IAV control (electronic supplementary material, figure S1).

## 4. Discussion

Exposure to airborne particulate matter has been widely shown to be associated with increased incidence and altered resolution of respiratory infections [5–7,15,16]. However, the mechanisms behind this susceptibility remain poorly understood. We have previously shown that polystyrene nanoparticles can interact with SP-A and SP-D and that this interaction can alter nanoparticle cellular uptake [30,37]. It was therefore hypothesized that nanoparticles would inhibit the ability of these collectins to neutralize viral challenges through sequestering the protein to the nanoparticle surface. Here, we show for the first time that nanoparticles can modulate the ability of the innate immune molecules SP-A and SP-D to neutralize *in vitro* viral infection. This modulation was dependent on the protein, nanoparticle, nanoparticle concentration and cell type under investigation. This could be an important step in establishing the mechanisms behind the increased susceptibility to infection following particle exposure.

This study evaluated the effect of unmodified and surface modified 100 nm polystyrene particles on the surfactant protein mediated neutralization of influenza infection in three cell lines. These nanoparticles were chosen as we have previously shown that SP-A and SP-D interact with these particles and result in differential uptake by alveolar macrophages [30,37]. The cell lines were chosen to reflect those found within the alveolus, namely TT1, A549 and THP-1 cells as models for ATI, ATII and macrophages, respectively. ATI cells are large, thin squamous cells that comprise 94% of the alveolar surface area but constitute only a third of the total number of alveolar epithelial cells [38]. ATII cells make up more than 60% of the number of epithelial cells within the alveolus and are responsible for the production and recycling of pulmonary surfactant [39]. A549 cells were derived from a lung adenocarcinoma and were originally considered to be a model for ATII cells [40–42]. However, other investigators have shown that as they do not possess many of the typical ATII phenotypic characteristics, such as surfactant production or alkaline phosphatase activity, they are not a good ATII model [36,43,44]. Despite these findings, A549 cells are one of the main cell types used to investigate the toxicity of nanoparticles on alveolar epithelial cells and have therefore been included in this study [45–49].

Low concentrations of unmodified polystyrene nanoparticles resulted in a reduction in the ability of surfactant proteins to neutralize IAV infection when incubated with SP-A in A549 cells or SP-D in TT1 cells, whereas at higher U-PS concentrations an increase in the surfactant protein mediated IAV neutralization was reported. Interestingly, low concentrations of A-PS also inhibited the neutralization of IAV infection by SP-A in TT1 cells and with SP-D in A549 cells (i.e. the reciprocal to the effect with U-PS). This differential effect of nanoparticle concentration on the modulation of surfactant protein mediated IAV neutralization by A-PS and U-PS could suggest a protein sequestration mechanism at low particle concentrations and a particle agglomeration mechanism at higher concentrations. As the amount of particles in the *in vitro* system increase, the amount of ‘available protein’ in solution will decrease as the particles sequester the surfactant protein. As the concentration of nanoparticle increases further, the effect of protein sequestration on influenza infection is minimized and then reversed by the agglomeration of nanoparticle and influenza complexes. This shows that low *in vitro* nanoparticle concentrations can lead to deficiencies in SP-A and SP-D, which in turn can enhance the susceptibility to influenza infection. The implications of this could extend far beyond virus neutralization by these collectins. The functions of SP-A and SP-D are multifaceted and the perturbation of their function has been linked to the pathogenesis of a number of diseases (e.g. chronic obstructive pulmonary disease (COPD) and idiopathic pulmonary fibrosis). Further work is necessary to determine the effect of nanoparticles on the other functions of SP-A and SP-D.

The highest concentration of U-PS particles (5 cm^2^ ml^−1^) tended to enhance the IAV neutralizing activity of both SP-A and SP-D in each of the three cell lines studied, except for SP-A with THP-1 cells. In order to establish whether this was due to the enhanced agglomeration of the nanoparticle–protein complex or through direct interactions of the complex with the cells under investigation, a haemagglutination inhibition assay was conducted. The results of this assay showed that at the highest concentration studied, the nanoparticles themselves, in the absence of protein and virus, could result in the apparent haemagglutination of red blood cells (data not shown). We therefore cannot exclude that, at the 5 cm^2^ ml^−1^ concentration, U-PS particles can interact directly with cells and this may, by squelching the system with high concentrations of nanoparticles, contribute to the observed enhanced protective effect of the surfactant proteins. The concentration of 5 cm^2^ ml^−1^ would therefore be the upper limit of U-PS concentrations that could be tested in *in vitro* infection models.

In a previous study, acute high doses of carbon black resulted in a protective effect in mice against *Streptococcus pneumonia* [50]. The dose used in that study, however, was 1000 µg/mouse given in two equal installations 3 days apart and represents a dose far in excess of environmentally relevant concentrations. This study shows that concentration is a critical factor in determining the effect of nanoparticles in infection models and that acute doses are not representative of chronic or low dose effects.

BSA was used in this study to examine the effect of a non-specific protein corona on IAV infection rates. This was done to determine whether any effects observed with surfactant proteins were due to surfactant protein specific effects or due to the presence of protein. BSA had no significant effect on IAV infection in any of the cells studied. The incubation of U-PS particles with BSA had no significant effect on IAV infection at any of the concentrations or cell lines tested.

A-PS particles resulted in a reduction in IAV infection at the highest concentration studied in A549 and TT1 cells. This was not attributable to cell toxicity at this concentration as no reduction in cell viability was observed using the MTT assay (data not shown). Amine particles have been proposed to act as a proton sponge within the lysosome, resulting in enhanced proton pump activity, lysosomal swelling and rupture [51]. The acidification of the endosome is an important step in virus entry as it initiates the conformational change in the HA and the fusion of viral and endosomal membranes [52,53]. This could explain the reduction of IAV infection at the highest A-PS concentration studied, as the amine particles could be perturbing the acidification of the endosome and inhibiting IAV release and replication within the cell. However, other mechanisms may also be involved. For instance, the envelope of the IAV virion is derived from the host cellular membrane. Amine particles have been shown to bind anionic patches on cell membranes and this interaction has been shown to cause membrane disruptions [54,55]. It is therefore possible that A-PS particles could be binding to the lipid virion envelope and either disrupting the integrity of the virion or sterically hindering its attachment to the cell membrane. As the A-PS particles self-agglomerate at physiological temperatures, the IAV could become entrapped within these agglomerates, thereby inhibiting virus attachment and entry into the cell [37]. This may be the reason that the effect of high concentrations of A-PS on IAV infection is less pronounced with the THP-1 cells, as phagocytes preferentially internalize larger particles [56]. Interestingly, in preliminary experiments high concentrations of U-PS particles (50 cm^2^ ml^−1^) were shown to inhibit IAV infection in A549 cells (data not shown). Gold nanoparticles have also been previously shown to inhibit HIV-1 infection through binding the surface viral glycoprotein (gp120) and inhibiting its attachment with CD4 cells [57]. The binding of these polystyrene nanoparticles to HA should therefore be investigated.

This study used four concentrations of nanoparticles over a 3125-fold dilution range. These concentrations were chosen in order to establish the effect of low and high doses of nanoparticles in the developed *in vitro* system. While this concentration range showed nanoparticle modulation of SP-A and SP-D activity, it also showed that this modulation was highly susceptible to nanoparticle concentration with perhaps only a narrow window of efficacy. In order to establish the extent of the nanoparticle modulatory concentration range further, *in vitro* work using this system is required.

Individuals with existing respiratory conditions such as COPD show reduced levels of pulmonary SP-A and SP-D levels due to their translocation to the systemic circulation. These individuals show enhanced susceptibility to infections, especially following enhanced PM exposure. The sequestration of SP-A and SP-D by particles within the alveolus could therefore play an important role in the pathogenesis of this susceptibility. The results from this study show that nanoparticles can sequester surfactant proteins and that this sequestration can reduce surfactant protein function. However, there were a number of factors which affected the ability of the nanoparticle to alter protein function. A key determining factor was the concentration of the nanoparticles used in the study. Surfactant protein function was only impinged at the lower two concentrations used in this study. At higher concentrations, either no effect was observed or nanoparticles enhanced surfactant protein activity. Many toxicity studies use high acute doses to determine the toxicokinetics of exogenous substances; this practice seems to be particularly prevalent in nanotoxicology. However, this study shows fundamental differences between high (acute) and low (chronic) concentrations. The highest concentration used in this study was 5 cm^2^ ml^−1^ which is equivalent to approximately 8.8 µg ml^−1^. Even this dose is modest compared with other *in vitro* nanotoxicology studies, which typically range between 10 and 300 µg ml^−1^ [54,58–61]. The low concentrations used this study therefore represent several times less than those used in conventional nanotoxicology studies. The low doses used in this study (0.0028 and 0.07 µg ml^−1^ for 0.0016 and 0.04 cm^2^ ml^−1,^ respectively) are more likely to represent chronic exposure whereby the surfactant system is gradually depleted, rather than acute concentrations where the surfactant is rapidly overwhelmed.

These results demonstrate the difficulties in using acute high concentrations *in vitro* and extrapolating the results towards environmentally relevant concentrations associated with chronic exposure. This model is the first step in developing a chronic *in vitro* cell culture exposure system. In future, the model will be developed further to determine the effect of chronic long-term pre-exposure to nanoparticles on influenza infection and surfactant protein mediated neutralization. This study highlights the urgent need for further investment and research into SP-A and SP-D sequestration by nanoparticles and the resulting effects of this sequestration on protein function.

## Supplementary Material

Supplementary Material
