# Supplementary material for: Nanoparticles modulate surfactant protein A and D mediated protection against influenza A infection in vitro
Source: Philos Trans R Soc Lond B Biol Sci. 2015 Feb 5;370(1661):20140049. doi: 10.1098/rstb.2014.0049 (PMC4275912; doi:10.1098/rstb.2014.0049)
Supplement: Supplementary Material [file rstb20140049supp1.pdf]

# Electronic Supplementary Material

## Nanoparticles modulate Surfactant Protein A and D mediated protection against Influenza A infection *in vitro*.

**DOI 10.1098/rstb.2014.0049**

Zofi McKenzie, Michaela Kendall, Rose-Marie Mackay, Teresa D. Tetley, Cliff Morgan, Mark Griffiths, Howard W. Clark and Jens Madsen

| **Treatment** | **NP concentration (cm²/mL)** | | | | | |
|---|---|---|---|---|---|---|
| **A549 cells** | | **0** | **0.0016** | **0.04** | **1** | **5** |
| U-PS | Mean | 100.0 | 100.4 | 97.0 | 98.7 | 100.2 |
| | p value | - | 0.219 [b] | 0.219 [b] | 0.219 [b] | 1.000 [b] |
| U-PS + SP-A | Mean | 65.5 | 73.5 | 74.1 | 63.4 | 48.8 |
| | p value | **0.019 [a]** | **0.050** [b] | **0.050** [b] | 0.275 [b] | **0.050** [b] |
| U-PS + SP-D | Mean | 44.7 | 48.6 | 44.2 | 45.3 | 13.9 |
| | p value | **0.014 [a]** | 0.386 [b] | 0.773 [b] | 1.000 [b] | **0.021** [b] |
| U-PS + BSA | Mean | 101.8 | 108.6 | 100.3 | 99.1 | 98.8 |
| | p value | 0.435 [a] | 0.275 [b] | 0.827 [b] | 0.827 [b] | 0.513 [b] |
| **TT1 cells** | | | | | | |
| U-PS | Mean | 100.0 | 98.4 | 92.7 | 92.2 | 95.0 |
| | p value | - | 0.487 [b] | **0.037** [b] | 0.487 [b] | 0.487 [b] |
| U-PS + SP-A | Mean | 73.6 | 67.7 | 63.8 | 74.4 | 60.6 |
| | p value | **0.037 [a]** | 0.513 [b] | 0.513 [b] | 0.827 [b] | 0.127 [b] |
| U-PS + SP-D | Mean | 60.1 | 69.1 | 78.8 | 59.7 | 16.3 |
| | p value | **0.037 [a]** | 0.513 [b] | **0.050** [b] | 0.827 [b] | **0.050** [b] |
| U-PS + BSA | Mean | 99.7 | 92.5 | 94.0 | 88.1 | 101.7 |
| | p value | 0.487 [a] | 0.513 [b] | 0.827 [b] | 0.513 [b] | 0.827 [b] |
| **THP-1 cells** | | | | | | |
| U-PS | Mean | 100.0 | 112.4 | 105.6 | 106.3 | 102.0 |
| | p value | - | 0.219 [b] | 0.219 [b] | 0.219 [b] | 1.000 [b] |
| U-PS + SP-A | Mean | 90.0 | 97.0 | 101.5 | 91.5 | 98.1 |
| | p value | **0.014 [a]** | 0.564 [b] | 0.149 [b] | 0.386 [b] | 0.564 [b] |
| U-PS + SP-D | Mean | 68.4 | 70.7 | 75.6 | 67.1 | 46.4 |
| | p value | **0.014 [a]** | 0.386 [b] | 0.386 [b] | 0.773 [b] | **0.021** [b] |
| U-PS + BSA | Mean | 111.7 | 109.2 | 86.2 | 103.9 | 90.6 |
| | p value | 0.435 [a] | 0.827 [b] | 0.275 [b] | 0.827 [b] | 0.275 [b] |

**Table S1: Relative Infection following influenza pre-incubation with U-PS and proteins.** [a]p values determined using Mann-Whitney U test versus protein free control; [b]p values determined using Mann-Whitney U test versus nanoparticle free protein containing control.

| **Treatment** | | **NP concentration ($cm^2$/mL)** | | | | |
|---|---|---|---|---|---|---|
| **A549 cells** | | **0** | **0.0016** | **0.04** | **1** | **5** |
| A-PS | Mean | 100.0 | 98.0 | 96.0 | 92.6 | 66.8 |
| | p value | - | 1.000 [b] | 0.219 [b] | 0.219 [b] | **0.014** [b] |
| A-PS + SP-A | Mean | 53.3 | 52.5 | 53.6 | 60.6 | 46.8 |
| | p value | **0.019** [a] | 0.513 [b] | 0.827 [b] | 0.513 [b] | 0.513 [b] |
| A-PS + SP-D | Mean | 37.6 | 45.7 | 42.5 | 42.5 | 33.3 |
| | p value | **0.019** [a] | **0.043** [b] | 0.248 [b] | 0.248 [b] | 0.248 [b] |
| A-PS + BSA | Mean | 101.1 | 110.1 | 102.9 | 101.5 | 82.0 |
| | p value | 0.219 [a] | 0.513 [b] | 0.827 [b] | 0.827 [b] | 0.275 [b] |
| **TT1 cells** | | | | | | |
| A-PS | Mean | 100.0 | 102.5 | 101.2 | 102.5 | 80.2 |
| | p value | - | 0.577 [b] | 0.095 [b] | 0.577 [b] | **0.005** [b] |
| A-PS + SP-A | Mean | 63.0 | 69.0 | 74.0 | 69.7 | 57.3 |
| | p value | **0.007** [a] | 0.248 [b] | **0.021** [b] | 0.386 [b] | 0.386 [b] |
| A-PS + SP-D | Mean | 60.9 | 54.0 | 55.2 | 55.5 | 46.6 |
| | p value | **0.016** [a] | 0.251 [b] | 0.602 [b] | 0.465 [b] | 0.076 [b] |
| A-PS + BSA | Mean | 96.6 | 105.2 | 101.7 | 97.9 | 89.0 |
| | p value | 0.180 [a] | 0.117 [b] | 0.465 [b] | 0.917 [b] | 0.602 [b] |
| **THP-1 cells** | | | | | | |
| A-PS | Mean | 100.0 | 101.4 | 99.9 | 101.9 | 96.2 |
| | p value | - | 0.219 [b] | 1.000 [b] | 1.000 [b] | 0.219 [b] |
| A-PS + SP-A | Mean | 72.0 | 80.2 | 72.1 | 68.5 | 74.4 |
| | p value | **0.019** [a] | 0.127 [b] | 0.827 [b] | 0.827 [b] | 0.827 [b] |
| A-PS + SP-D | Mean | 63.1 | 69.4 | 60.5 | 64.3 | 64.1 |
| | p value | **0.014** [a] | 0.386 [b] | 0.885 [b] | 1.000 [b] | 0.885 [b] |
| A-PS + BSA | Mean | 102.0 | 108.2 | 103.5 | 84.2 | 100.6 |
| | p value | 0.435 [a] | 0.513 [b] | 0.827 [b] | 0.513 [b] | 0.513 [b] |

**Table S2: Relative Infection following influenza pre-incubation with A-PS and proteins.** [a]p values determined using Mann-Whitney U test versus protein free control; [b]p values determined using Mann-Whitney U test versus nanoparticle free protein containing control. Significant values in bold.

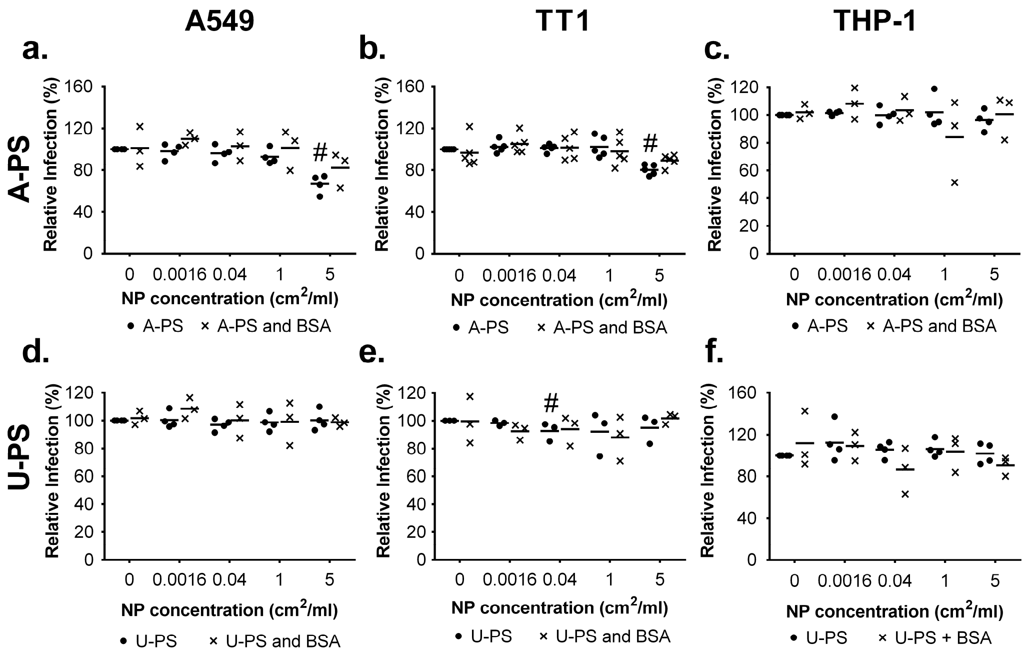


**Figure S1: Effect of 100 nm A-PS and U-PS with BSA on influenza A infection**. A-PS were pre-incubated with 5 µg/mL BSA prior to incubation with IAV and treatment of **A**. A549, **B**. TT1 and **C**. differentiated THP-1 cells. U-PS particles were pre-incubated with 5 µg/mL BSA prior to incubation with IAV and treatment of **D**. A549 **E**. TT1 and **F**. THP-1 cells. Horizontal line represents mean of at least three independent experiments conducted in duplicate. Statistics determined using Mann-Whitney U test where *$p \leq 0.050$ compared to relative infection in particle free protein control; #$p \leq 0.050$ versus IAV alone.
